# Manual of the Diseases of the Eye

**Published:** 1904-02

**Authors:** 


Manual of the Diseases of the Eye. For Students and General Practitioners. By Charles H. May, M. D., Chief of Clinic and Instructor in Ophthalmology, College of Physicians and Surgeons, Medical Department, Columbia University, New York. Third edition, revised. Duodecimo, 421 pages, with 275 original illustrations, including 16 plates, with 36 colored figures. New York: William Wood & Co. 1903. (Price, $2.00.)

Some slight changes have been made in this edition. The book was so carefully prepared in the first instance, that scarcely anything could be added which would improve its value as an aid in diagnosticating and treating most diseases of the eye. The elimination of the confusing grouping of remedies usually employed is to be commended. The only criticism we feel justified in offering is one that applies to all textbooks on this subject,—namely, the limited therapeutic hints on lid treatment. With this exception no fault can be found, for it is one of the best reference books for students and practitioners yet issued.

